# Supplementary material for: Predictors of Mortality in Patients with Advanced Cancer—A Systematic Review and Meta-Analysis
Source: Cancers (Basel). 2022 Jan 11;14(2):328. doi: 10.3390/cancers14020328 (PMC8774229; doi:10.3390/cancers14020328)
Supplement: Supplementary file 1 [file cancers-14-00328-s001.zip › cancers-1512331-supplementary.pdf]

Supplementary Material

Predictors of mortality in patients with advanced cancer – A systematic review and meta-analysis

Table S1. Search strategy for the online databases

Embase<br>**(**'chronic disease'/de OR 'lung fibrosis'/exp OR 'obstructive airway disease'/exp OR 'neoplasm'/exp OR (((chronic* OR advance* OR palliative) NEAR/4 (ill* OR diseas* OR condition* OR lung OR pulmonar* OR cancer* OR neoplas* OR tumor* OR tumour* OR patient*)) OR ((lung OR pulmonar* OR airway) NEAR/3 (fibros* OR obstruct*)) OR COPD OR asthma* OR bronchitis):ab,ti**) AND** ('physical disease by body function'/de OR 'health status indicator'/exp OR 'cognition'/de OR 'hospital admission'/de OR 'hospital readmission'/de OR (condition OR conditions OR cognit* OR admission* OR readmission* OR ((symptom*) NEAR/3 (sign* OR digest* OR respirat* OR pathol*))):ab,ti) **AND** ('terminal care'/exp OR 'death'/exp OR ('end of life' OR ((mors* OR mortalit* OR died OR die OR dying OR death OR fatal*) NEAR/6 (year OR month OR months OR week OR weeks OR days OR time*)) OR ((end *OR EOL* OR palliat* OR terminal* OR final OR last) NEAR/3 (care OR phase* OR stage* OR month* OR week* OR year))):ab,ti) **AND** ('prediction'/de OR 'predictive value'/de OR 'prognosis'/exp OR 'prognostic assessment'/exp OR ('surprise question' OR ((predict* OR indicat* OR prognost*) NEAR/4 (value* OR factor* OR tool* OR index OR indices OR assess*)) OR ((early OR time*) NEXT/1 (identificat*))):ab,ti) **AND** english:la **AND (**'article'/it OR 'article in press'/it OR 'note'/it OR 'review'/it**)**
MEDLINE<br>**(**"Chronic Disease"/ OR exp "Pulmonary Fibrosis"/ OR exp "Lung Diseases, Obstructive"/ OR exp "Neoplasms"/ OR (((chronic* OR advance* OR palliative) ADJ4 (ill* OR diseas* OR condition* OR lung OR pulmonar* OR cancer* OR neoplas* OR tumor* OR tumour* OR patient*)) OR ((lung OR pulmonar* OR airway) ADJ3 (fibros* OR obstruct*)) OR COPD OR asthma* OR bronchitis).ab,ti.**) AND** ("Signs and Symptoms"/ OR exp "Health Status Indicators"/ OR "Cognition"/ OR exp "Hospitalization"/ OR (condition OR conditions OR cognit* OR admission* OR readmission* OR ((symptom*) ADJ3 (sign* OR digest* OR respirat* OR pathol*))).ab,ti.) **AND** (exp "Terminal Care"/ OR exp "Death"/ OR ("end of life" OR ((mors* OR mortalit* OR died OR die OR dying OR death OR fatal*) ADJ6 (year OR month OR months OR week OR weeks OR days OR time*)) OR ((end *OR EOL* OR palliat* OR terminal* OR final OR last) ADJ3 (care OR phase* OR stage* OR month* OR week* OR year))).ab,ti.) **AND** ("Predictive Value of Tests"/ OR "Prognosis"/ OR ("surprise question" OR ((predict* OR indicat* OR prognost*) ADJ4 (value* OR factor* OR tool* OR index OR indices OR assess*)) OR ((early OR time*) ADJ1 (identificat*))).ab,ti.) **AND** English.lg. **AND** Journal Article.pt.
Cochrane Central<br>**(**(((chronic* OR advance* OR palliative) NEAR/4 (ill* OR diseas* OR condition* OR lung OR pulmonar* OR cancer* OR neoplas* OR tumor* OR tumour* OR patient*)) OR ((lung OR pulmonar* OR airway) NEAR/3 (fibros* OR obstruct*)) OR COPD OR asthma* OR bronchitis):ab,ti**) AND** ((condition OR conditions OR cognit* OR admission* OR readmission* OR ((symptom*) NEAR/3 (sign* OR digest* OR respirat* OR pathol*))):ab,ti) **AND** (('end of life' OR ((mors* OR mortalit* OR died OR die OR dying OR death OR fatal*) NEAR/6 (year OR month OR months OR week OR weeks OR days OR time*)) OR ((end *OR EOL* OR palliat* OR terminal* OR final OR last) NEAR/3 (care OR phase* OR stage* OR month* OR week* OR year))):ab,ti) **AND** (('surprise question' OR ((predict* OR indicat* OR prognost*) NEAR/4 (value* OR factor* OR tool* OR index OR indices OR assess*)) OR ((early OR time*) NEXT/1 (identificat*))):ab,ti)
Web of Science<br>**TS=((**((((chronic* OR advance* OR palliative) NEAR/3 (ill* OR diseas* OR condition* OR lung OR pulmonar* OR cancer* OR neoplas* OR tumor* OR tumour* OR patient*)) OR ((lung OR pulmonar* OR airway) NEAR/2 (fibros* OR obstruct*)) OR COPD OR asthma* OR bronchitis)**) AND** ((condition OR conditions OR cognit* OR admission* OR readmission* OR ((symptom*) NEAR/2 (sign* OR digest* OR respirat* OR pathol*)))) **AND** (("end of life" OR ((mors* OR mortalit* OR died OR die OR dying OR death OR fatal*) NEAR/6 (year OR month OR months OR week OR weeks OR days OR time*)) OR ((end *OR EOL* OR palliat* OR terminal* OR final OR last) NEAR/2 (care OR phase* OR stage* OR month* OR week* OR year)))) **AND** (("surprise question" OR ((predict* OR indicat* OR prognost*) NEAR/2 (value* OR factor* OR tool* OR index OR indices OR assess*)) OR ((early OR time*) NEAR/1 (identificat*))))**) AND LA=** English **AND DT=**Article
PubMed<br>**(**((chronic*[tiab] OR advance*[tiab] OR palliative[tiab]) AND (ill[tiab] OR illness[tiab] OR diseas*[tiab] OR condition*[tiab] OR lung[tiab] OR pulmonar*[tiab] OR cancer*[tiab] OR neoplas*[tiab] OR tumor*[tiab] OR tumour*[tiab] OR patient[tiab] OR patients[tiab])) OR ((lung[tiab] OR pulmonar*[tiab] OR airway[tiab]) AND (fibros*[tiab] OR obstruct*[tiab])) OR COPD[tiab] OR asthma*[tiab] OR bronchitis[tiab]**) AND (**condition[tiab] OR conditions[tiab] OR cognit*[tiab] OR admission*[tiab] OR readmission*[tiab] OR ((symptom*[tiab]) AND (sign[tiab] OR signs[tiab] OR digest*[tiab] OR respirat*[tiab] OR pathol*[tiab])))**) AND (**end of life[tiab] OR ((mors*[tiab] OR mortalit*[tiab] OR died[tiab] OR die[tiab] OR dying[tiab] OR death[tiab] OR fatal*[tiab]) AND (year[tiab] OR month[tiab] OR months[tiab] OR week[tiab] OR weeks[tiab] OR days[tiab] OR time*[tiab])) OR ((end[tiab] *OR EOL*[tiab] OR palliat*[tiab] OR terminal*[tiab] OR final[tiab] OR last[tiab]) AND (care[tiab] OR phase*[tiab] OR stage*[tiab] OR month*[tiab] OR week*[tiab] OR year[tiab])))**) AND (**surprise question[tiab] OR ((predict*[tiab] OR indicat*[tiab] OR prognost*[tiab]) AND (value*[tiab] OR factor*[tiab] OR tool*[tiab] OR index[tiab] OR indices[tiab] OR assess*[tiab])) OR early identif*[tiab] OR timely identif*[tiab]**) AND** publisher[sb]

Table S2. Domains and items for risk-of-bias assessment

Domain	Items	Total domain points
Study participation	1. Recruitment procedure<br>2. Study population<br>3. In- and exclusion criteria<br>4. Baseline study characteristics	8
Study attrition	5. Number of loss to follow-up<br>6. Reasons for loss to follow-up<br>7. Differences between completers and non-completers<br>8. Handling of missing data	8
Predictor measurement	9. Definition of predictor(s)<br>10. Measurement of predictor(s)<br>11. Handling of predictor(s)<br>12. Data presentation	8
Outcome measurement	13. Outcome definition<br>14. Outcome presentation	4
Statistical analysis and confounding	15. Method of selection of variables<br>16. Occurrence of mortality in relation to predictors<br>17. Presentation of crude variables (univariate analysis)<br>18. Presentation of adjusted variables (multivariate analysis)<br>19. Account for potential confounders<br>20. Selective reporting	12
Model performance	21. Discriminative ability<br>22. Internal validation<br>23. External validation	6

Each domain is scored as low risk-of-bias (≥80% of the total domain points), moderate risk-of-bias (60-79% of the total domain points), or high risk-of-bias (< 60% of the total domain points).

Table S3. Hazard ratios for mortality based on studies with low risk-of-bias

Predictor	N studies	N patients	Pooled HR (95% CI)
Age (per 10-year increase)	2[1,2]	782	1.22 (1.11–1.34)
Male sex (ref: female)	2[1,3]	1870	1.21 (1.08–1.35)
ECOG performance status 1+ (ref: 0–1)	2[1,4]	1154	2.22 (0.98–5.03)

CI: confidence interval; HR: hazard ratio; ref: reference; ECOG: Eastern Cooperative Oncology Group.

Table S4. Variables not included in the meta-analysis

Variable group	Variable	Number of studies
**Age**	• Age at bone metastases diagnosis >50 vs. ≤50 years<br>• Age <50 vs. ≥60 years<br>• Age 50–59 vs. ≥60 years<br>• Age ≤60 vs. >60<br>• Age >64 vs. ≤64 years<br>• Age ≥67 vs. <67 years<br>• Age 75–79 vs. <75 years<br>• Age ≥80 vs. <75 years	6[5-10]
**Body mass index**	• BMI kg/m2 Overweight (BMI >25) vs. normal (BMI 18.5–25)<br>• BMI kg/m2 Underweight (BMI <18.5) vs. normal (BMI 18.5–25)<br>• BMI <23 vs. ≥23 kg/m2	2[8,9]
**Use of medication**	• No of drugs<br>• ACEi<br>• Beta-blocker<br>• Dexamethasone<br>• Diuretic<br>• Megestrol acetate<br>• Use of morphine<br>• Tamoxifen	2[11,12]
**Comorbidity (score)**	• Comorbidity Index CIRS (cont. Var)<br>• Charlson score 1 vs. 0<br>• Charlson score 2 vs. 0<br>• Charlson score 1+ (ref 0)<br>• Moderate to severe comorbidity vs. absent/mild<br>• ASA (indicating comorbidity at time of surgery) score >1 (with comorbidity) vs. 1 (without comorbidity<br>• ASA score (<3 or ≥3)	6[8,10,12-15]
**Cardiovascular comorbidity**	• Atrial fibrillation<br>• Vascular disease as co-morbidity present vs. absent<br>• IHD: ischemic heart disease<br>• NYHA class<br>• Hypertension<br>• Diabetes<br>• Higher LVEF	1[11]
**Other comorbidity**	• COPD<br>• Syndromes yes vs. no<br>• Psychiatric diseases yes vs. no	1[11,12]
**Performance status**	• ECOG performance status<br>• Karnofsky PS 80–90% vs. normal (ECOG PS 0–1 vs. 0)<br>• Karnofsky PS 50–70 vs. 80–100% (ECOG PS 1–2 vs. 0–1)<br>• Karnofsky PS 70–80 vs. ≤60 (ECOG PS 1 vs. 2+)<br>• ECOG PS 2 vs. 1<br>• Karnofsky performance score ≤60 vs. >60 (ECOG PS 2+ vs. 0–1)<br>• Initial ECOG PS 2–3 vs. 0<br>• Performance status 2–3 vs. 0–1<br>• Performance status ECOG 2–4 vs. 0–1<br>• Karnofsky PS 30–40 vs. 90% (ECOG PS 3 vs. 0)<br>• ECOG Performance status 3+ versus 0<br>• Performance status 3–4 vs. 0–2<br>• Karnofsky PS <50% (ECOG PS 3+ vs. unknown)	13[6,7,12,13,16-24]
**Activities of daily living**	• Instrumental Activities of daily living (IADL)<br>• IADL (instrumental activities of daily living)<br>• iADL Intermediate vs. Better<br>• iADL Worse vs. Better<br>• Dependence in instrumental activities of daily living (Lawton scale <20/24)<br>• Activities of daily living (ADL)<br>• ADL One or more dependence vs. no dependence	4[10,12,17,25]
**Mobility**	• Ambulation status With support or nonambulartory vs. Without support<br>• Timed Get Up and Go Impaired vs. No impairments (≤ 20 seconds)<br>• Usual gait speed (UGS): assessed over a 4-m course starting from a still position<br>• Short physical performance battery (SPPB): chair-stand, balance and walking speed test<br>• Walking ability 4–8 months after admission: unable to walk vs. able<br>• 10 meter walk test (10MWT) at admission<br>• Bergs Balance Scale (BBS) at admission	4[17,26-28]

**Handgrip strength**	• Handgrip strength<br>• Handgrip 25th percentile<br>• Handgrip ≤10 percentile	2[17,29]
**Tumor stage**	• Stage of cancer<br>• Tumor stage advanced (metastatic) vs. localized (nonmetastatic)<br>• Cancer stage: more advanced (stages III and IV) vs. less advanced (stages I, II, and unknown)<br>• Stage at diagnosis IV (ref: locoregional disease stages I–III)<br>• Stage IV vs. IIIb<br>• Stage IV vs. III<br>• Severity of SCLC<br>• Severity of NSCLC<br>• Severity of breast cancer	7[3,10,11,28,30-32]
**Metastases**	• Visceral metastases<br>• Visceral or cerebral metastases<br>• Major organ metastases: unmovable vs. none<br>• ≥ 4 metastatic sites<br>• Metastatic spread to the brain<br>• Number of brain metastases 1 vs. >3<br>• Number of brain metastases 2–3 vs. >3<br>• Extra-cranial metastatic disease Yes vs. No<br>• Pleural metastasis<br>• Pleural effusion Yes vs. No<br>• Extrahepatic spread present vs. absent<br>• Leptomeningeal disease yes vs. no<br>• Cervical location of spinal metastasis (symptomatic metastases in cervical spine)<br>• Pathological fracture<br>• Site of metastases Others vs. Bone only<br>• Multiple skeletal metastasis<br>• Number of bone metastases 1–3 vs. >3<br>• Location of skeletal metastases 'appendicular and axial bone' vs. appendicular bone<br>• Location of skeletal metastases axial bone vs. appendicular bone<br>• No. Of bone lesions on initial bone scan 5–15/ >15/superscan vs. 0–4	13[5-7,16,18,19,21,24,33-37]
**Ascites**	• Presence of ascites<br>• Persistent ascites	2[38,39]
**Portal vein thrombosis**	• Presence of portal vein thrombosis<br>• Portal vein thrombosis<br>• Thrombosis	3[22,38,39]
**Antitumor therapy**	• Treatment before baseline Yes/No<br>• Prior treatment history (previously treated as reference)<br>• No previous lung surgery<br>• Cancer treatment yes vs. no<br>• Chemotherapy Yes vs. No<br>• Previous chemotherapy<br>• Pre-operative chemotherapy present vs. absent<br>• Cancer treatment intent (palliative vs. curative)<br>• Curatively intended treatment of primary tumor<br>• Palliative care vs. Chemotherapy<br>• Palliative care vs. Sequential therapy<br>• Palliative care vs. TKI (EGFR tyrosine kinase inhibitors)<br>• Pembrolizumab after chemotherapy failure No vs. Yes<br>• Without treatment vs. Surgery, Palliative therapy (chemotherapy or radiotherapy), and Combined surgery with chemotherapy<br>• Radiotherapy Yes vs. No<br>• Treatment EBRT (conventional radiotherapy) yes vs. no	14[1,3,7,9,12,15,16,24,25,31,33,36,40,41]
**Weight change**	• Fat-free mass (per kg increase)<br>• Cachexia<br>• Weight loss<br>• Weight loss >8.1 kg vs. ≤8.1 kg<br>• Rate of weight loss >2,75% per month<br>• Baseline Weight Loss >11 lbs. vs. ≤10 lbs.<br>• Lung cancer by weight loss >8.1 kg<br>• Weight change ≥ -14% vs. -1.9 to 1.9%<br>• Weight change -2 to -13.9% vs. -1.9 to 1.9%<br>• Weight change >2% vs. -1.9 to 1.9%<br><br>• Body surface area (BSA) 0.5 unit increase	8[11,13,15,20,30,41-43]
**Nutrition / dietary intake**	• Mini Nutritional Assessment at risk/ poor nutrition, score ≤23.5 vs. Good nutrition, score >23.5	4[28,36,43,44]

	• Nutritional decay presence vs. absence<br>• Dietary intake normal food/reduced amount vs. normal food/normal amount<br>• Dietary intake Abnormal intake vs. normal food/normal amount<br>• Alcohol drinking Yes vs. No	
**Re-hospitalization**	• Inpatient [hospitalization] vs. outpatient<br>• Reason for admission (cardiac/pulmonary, neurologic, pain, failure to thrive vs. GI/genitourinary, abnormal labs, bleeding, infection)<br>• Readmission within 30 days<br>• Discharge with hospice services	2[25,44]
**Smoking**	• Smoking Yes vs. No	1[36]
**Social-related variables**	• Income quintile 1 (low) vs. 5<br>• Income quintile 2 vs. 5<br>• Income quintile 3 vs. 5<br>• Income quintile 4 vs. 5<br>• Caregiver yes vs. no	2[12,14]
**Dyspnea**	• Severity of dyspnea<br>• Dyspnea (Dyspnea at rest vs. No dyspnea)<br>• Shortness of breath mild vs. none<br>• Shortness of breath moderate vs. none<br>• Shortness of breath severe vs. none	3[11,21,45]
**Dysphagia**	• Dysphagia	1[43]
**Depression**	• Depression mild vs. none<br>• Depression moderate vs. none<br>• Depression severe vs. none	1[45]
**Nausea**	• Nausea mild vs. none<br>• Nausea moderate vs. none<br>• Nausea severe vs. none<br>• Moderate-to-severe nausea vs. absent-to-mild<br>• Nausea constant vs. absent	3[11,13,45]
**Appetite**	• Loss of appetite<br>• Appetite mild vs. none<br>• Appetite moderate vs. none<br>• Appetite severe vs. none	2[33,45]
**Fatigue**	• Fatigue mild vs. none<br>• Fatigue moderate vs. none<br>• Fatigue severe vs. none<br>• Fatigue constant vs. absent	2[11,45]
**Pain**	• Pain Present vs. Absent<br>• Pain mild vs. none<br>• Pain moderate vs. none<br>• Pain severe vs. none<br>• Severity of pain<br>• Subjective pain score from 0–10 (<6 vs. ≥6)	4[11,15,26,45]
**Neurological symptoms**	• Paresis Present vs. Absent<br>• Neurological deficits	2[7,26]
**Quality of life (domains)**	• QoL Intermediate vs. Better<br>• QoL Worse vs. Better<br>• HRQoL Anorexia<br>• QLQ-C30 scale QoL Domain Appetite loss<br>• HRQOL Appetite loss >0 vs. ≤0<br>• Psychosocial variable (higher score= better status) Eating appetite<br>• QLQ-C30 scale QoL Domain Cognitive<br>• QLQ-C30 scale QoL Domain Constipation<br>• QLQ-C30 scale QoL Domain Diarrhea<br>• QLQ-C30 scale QoL Domain Dyspnea<br>• HRQOL: Dysphagia (higher score= higher perception of symptom)<br>• QLQ-C30 scale QoL Domain Emotional<br>• QLQ-C30 scale QoL Domain Fatigue<br>• EORTC QLQ-C30 Fatigue<br>• HRQoL Role functioning<br>• FACT-G (Ch: higher score= better HRQoL) Functional well-being<br>• HRQOL Insomnia >33.3 vs. ≤33.3<br>• QLQ-C30 scale QoL Domain Nausea/vomiting<br>• QLQ-C30 scale QoL Domain Pain<br>• HRQOL: Pain (higher score= higher perception of symptom)<br>• QLQ-C30 scale QoL Domain Physical<br>• HRQoL Physical functioning<br>• FACT-G(Ch: higher score= better HRQoL) Physical well-being<br>• QLQ-C30 scale QoL Domain Role	8[2,3,10,19,30,31,37,46]

	• QLQ-C30 scale QoL Domain Social<br>• Baseline FACT-G Total score (increase in score)	
**a-Fetoprotein level**	• a-Fetoprotein level ≥400 ng/ml<br>• AFP >400 ng/mL	2[39,47]
**Albumin**	• Albumin <28 g/dL<br>• Albumin (per g/dL increase)<br>• Albumin <3.0 vs. ≥3.0 g/dl<br>• Serum albumin level <36 g/L by lymphocyte count <1x 109/L<br>• Serum albumin level <36 g/L by weight loss >8.1 kg<br>• Serum albumin level <35 g/L by time covariate	4[9,13,39,42]
**Alkaline phosphatase**	• Alkaline phosphatase<br>• ALP (alkaline phosphatase) >450 vs. ≤450 U/L	1[37]
**Bilirubin**	• Bilirubin >1.3 mg/dL vs. ≤1.3<br>• Bilirubin >50 mmol/L	2[39,40]
**C-reactive protein**	• C-reactive protein (mg/L)<br>• CRP ≥1.0 mg/dl<br>• CRP ≥5 mg/dl vs. <5 mg/dl<br>• C-reactive protein (>5 mg/l)<br>• CRP (C-reactive protein) >32 vs. ≤32 mg/l	5[2,9,20,23,47]
**Lactate dehydrogenase**	• Abnormal LDH (>240 U/L vs. ≤240 U/L)<br>• LDH (lactate dehydrogenase) >360 vs. ≤360 U/L<br>• LDH >618 U/L vs. ≤618 U/L	3[9,13,21]
**Lymfocyte**	• Lymphocyte count <1 x 109/L vs. ≥ 1 x 109/L<br>• Lymphocyte count <700 units/mm3 vs. ≥700<br>• Lymphocyte count <1600 vs. ≥1600 ul<br>• WBC count Abnormal (≥ 10.8 x 103/uL) vs. normal (<10.8)<br>• Elevated WBC<br>• Neutrophil-lymphocyte ratio ≥3 vs. <3	5[9,13,21,48,49]
**Platelets**	• Platelets >500,000 mm3 (upper limit of normal)<br>• Platelet <143 000/mm3	2[34,39]
**Serum sodium**	• Na (serum sodium)<br>• Natremia <135 mEq/l (lower limit of normal)	2[34,38]
**Other laboratory values**	• CA 19-9 ≥10 000 U/ml vs. < 10 000 U/ml<br>• Corrected calcium<br>• Creatinine >1.3 mg/dL<br>• Hemoglobin (continuous)<br>• Hyperglycemia<br>• High serum CEA (carcinoembryonic antigen) (unspecified)<br>• High serum CYFRA211 (cytokeratin-19-fragment) (unspecified)<br>• High serum NSE (neuron-specific enolase) (unspecified)<br>• High serum ProGRP (progastrin-releasing peptide) (unspecified)<br>• INR >1.2	5[4,11,23,36,39]
**Survival estimate**	• Clinical estimation of survival 2–6 mo. vs. ≤2 mo.<br>• Clinical estimation of survival >6 mo. vs. ≤2 mo.	1[13]
**Scales**	• Palliative Performance Scale (per each 10-point decrease)<br>• Palliative Prognostic Score (per point increase)<br>• Significant functional decay (PPS [Palliative Performance Scale] ≤ 50%)<br>• 15-item Geriatric Depression Scale >5 vs. ≤5<br>• GDS (≥5)<br>• MELD >10<br>• MPI 0.3 unit increase in MPI<br>• Cognitive impairment (Test of 9 or greater on the Short Blessed Test)	7[12,14,22,25,41,42,44]

Figure S1. Forest plots per individual predictor using random-effects model

Age (per 10-year increase)

Study	N patients	HR (95% CI)
Moss, 2010	826	1.06 (0.88–1.28)
Brunello, 2016	658	1.48 (0.96–2.28)
Fielding, 2007	358	1.17 (1.03–1.33)
Filippini, 2008	676	1.34 (1.22–1.48)
Scott, 2002	106	1.48 (1.09–2.00)
Seow, 2013	11,342	1.04 (1.02–1.07)
**Total (95% CI)** $I^2 = 85\%$		1.20 (1.05–1.38) p= 0.007

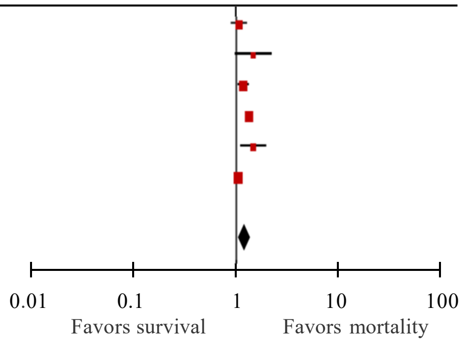

Male sex (ref: female)

Study	N patients	HR (95% CI)
Braun, 2011	1194	1.28 (1.11–1.49)
Soubeyran, 2012	348	2.40 (1.20–4.81)
Brunello, 2016	658	1.02 (0.65–1.60)
Jonna, 2016	803	1.23 (1.03–1.46)
Wei, 2019	71	3.99 (0.81–19.63)
Dharma-Wardene, 2004	42	3.12 (1.04–9.37)
Efficace, 2006	391	1.32 (1.03–1.69)
Filippini, 2008	676	1.11 (0.93–1.32)
Bartels, 2007	219	1.49 (1.05–2.13)
Katagiri, 2005	350	0.89 (0.67–1.18)
Maione, 2005	566	1.28 (0.92–1.69)
Seow, 2013	11,342	1.28 (1.20–1.37)
**Total (95% CI)** $I^2 = 39\%$		1.24 (1.14–1.36) p< 0.00001

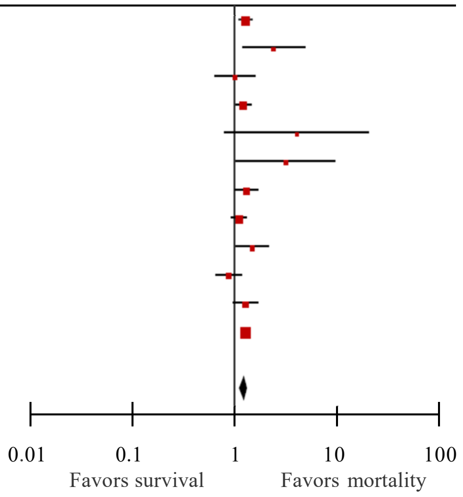

Disease stage III (ref: I)

Study	N patients	HR (95% CI)
Norman, 2010	399	10.81 (1.50–78.07)
Deans, 2007	220	2.88 (0.89–9.34)
Brunello, 2016	658	2.13 (0.85–5.31)
**Total (95% CI)** $I^2 = 7\%$		2.89 (1.42–5.90) p= 0.003

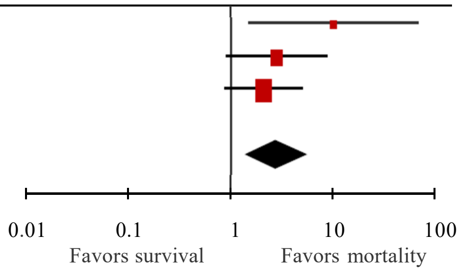

Disease stage IV (ref: I)

Study	N patients	HR (95% CI)
Norman, 2010	399	10.45 (1.38–79.36)
Deans, 2007	220	8.83 (2.70–28.84)
Brunello, 2016	658	6.69 (2.95–15.17)
**Total (95% CI)** $I^2 = 0\%$		7.58 (4.00–14.36) p< 0.00001

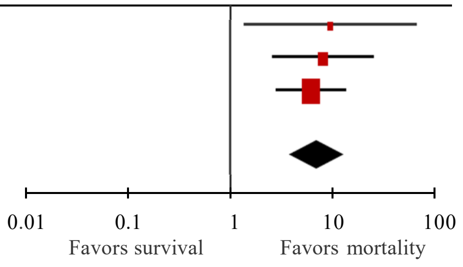

Continuation Figure S1

Liver metastases

**Study**	**N patients**	**HR (95% CI)**
Chow, 2008	395	0.47 (0.19–1.15)
Hoang, 2005	1436	1.32 (1.13–1.5)
Vigano, 2000	227	2.40 (1.65–3.50)
**Total (95% CI)** $I^2 = 86\%$		1.31 (0.71–2.39) p= 0.39

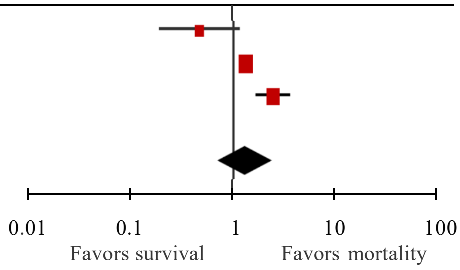

Karnofsky performance status

**Study**	**N patients**	**HR (95% CI)**
Cesari, 2013	200	1.42 (0.70–2.89)
Pointillart, 2011	142	0,98 (0.96–0.99)
Scott, 2002	106	0.96 (0.94–0.99)
**Total (95% CI)** $I^2 = 7\%$		0.97 (0.96–0.99) p= 0.0001

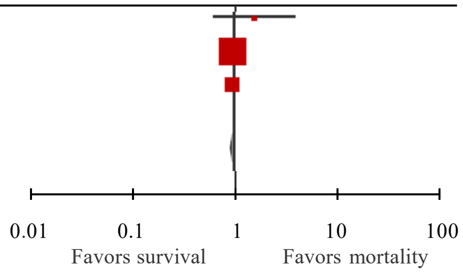

ECOG performance status 1 (ref: 0)

**Study**	**N patients**	**HR (95% CI)**
Collette, 2004	391	0.89 (0.64–1.24)
Deans, 2007	220	1.01 (0.65–1.58)
Brunello, 2016	658	3.12 (1.88–5.19)
Griguolo, 2018	668	1.43 (1.14–1.79)
Dharma-Wardene, 2004	42	0.49 (0.21–1.14)
Bartels, 2007	219	4.45 (1.53–12.93)
**Total (95% CI)** $I^2 = 82\%$		1.37 (0.88–2.13) p= 0.17

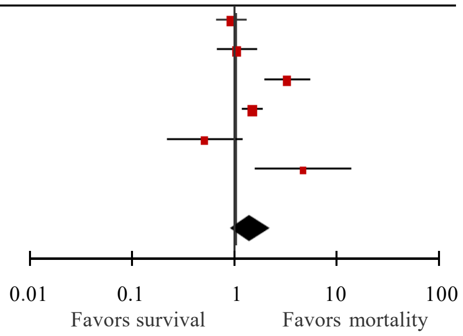

ECOG performance status 1+ (ref: 0–1)

**Study**	**N patients**	**HR (95% CI)**
Motzer, 2004	251	3.50 (2.09–5.87)
Penel, Hollebecque, 2008	119	1.92 (1.04–3.54)
Filippini, 2008	676	1.52 (1.26–1.82)
**Total (95% CI)** $I^2 = 78\%$		2.09 (1.23–3.56) p= 0.006

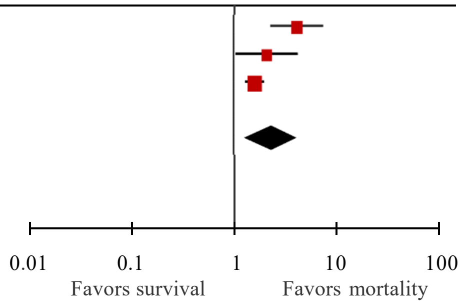

Continuation Figure S1

ECOG performance status 1–2 (ref: 0)

Study	N patients	HR (95% CI)
Deans, 2007	220	2.08 (1.26–3.43)
Hoang, 2005	1436	1.46 (1.26–1.70)
Bartels, 2007	219	5.67 (2.03–15.84)
**Total (95% CI)** $I^2 = 75\%$		2.13 (1.21–3.75) p= 0.009

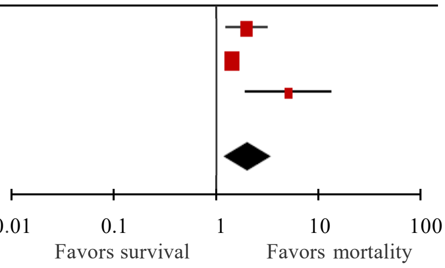

ECOG performance status 2 (ref: 0)

Study	N patients	HR (95% CI)
Marrero, 2005	244	14.29 (5.05–40.41)
Brunello, 2016	658	3.83 (1.78–8.24)
Griguolo, 2018	668	2.79 (2.06–3.78)
**Total (95% CI)** $I^2 = 78\%$		4.73 (2.06–10.85) p= 0.0002

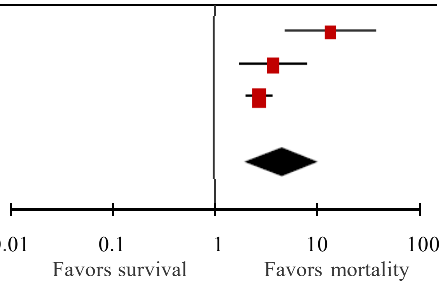

ECOG performance status 2+ (ref: 0)

Study	N patients	HR (95% CI)
Deans, 2007	220	4.83 (2.23–10.16)
Villa, 2011	285	2.50 (1.55–4.03)
Griguolo, 2018	668	5.48 (4.11–7.31)
**Total (95% CI)** $I^2 = 74\%$		4.06 (2.36–6.98) p< 0.00001

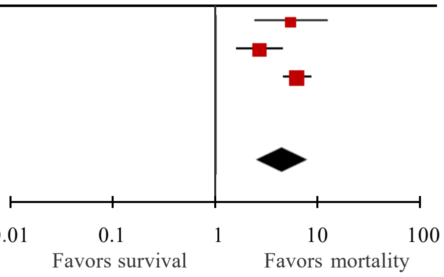

ECOG performance status 2 (ref: 0–1)

Study	N patients	HR (95% CI)
Park, 2016	403	1.53 (1.19–1.96)
Efficace, 2006	391	0.62 (0.39–0.96)
Maione, 2005	566	1.46 (1.13–1.89)
**Total (95% CI)** $I^2 = 85\%$		1.16 (0.74–1.82) p= 0.53

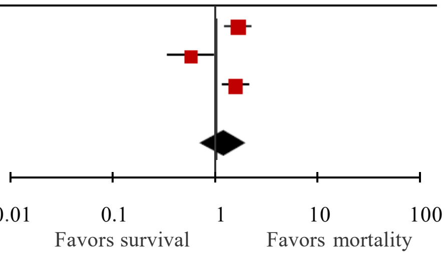

Continuation Figure S1

Charlson comorbidity index score 3+ (ref: 0)

Study	N patients	HR (95% CI)
Iversen, 2009	13,190	2.26 (1.92–2.66)
Lund, 2009	2315	1.72 (1.46–2.02)
Maione, 2005	566	0.84 (0.52–1.36)
**Total (95% CI)** $I^2 = 88\%$		1.60 (1.11–2.32) p= 0.01

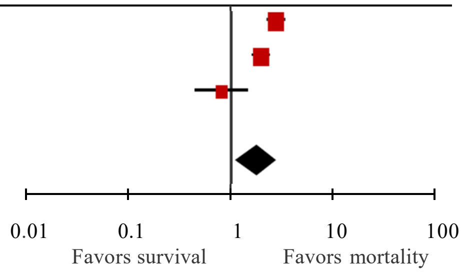

Lung cancer (ref: breast cancer)

Study	N patients	HR (95% CI)
Chow, 2008	395	2.33 (1.64–3.30)
Villa, 2011	285	1.57 (1.02–2.41)
Martin, 2010	1164	1.77 (1.37–2.29)
Seow, 2013	11,342	5.85 (4.83–7.09)
**Total (95% CI)** $I^2 = 96\%$		2.51 (1.24–5.06) p= 0.01

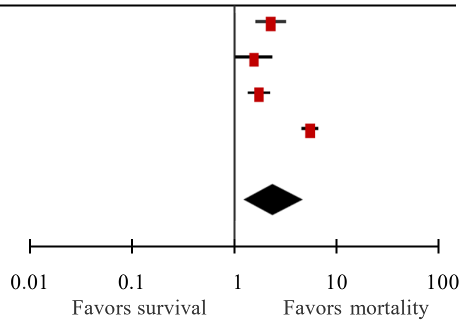

CI: confidence interval; HR: hazard ratio; mo.: months; N: number of; ECOG: Eastern Cooperative Oncology Group.

Figure S2. Funnel plots

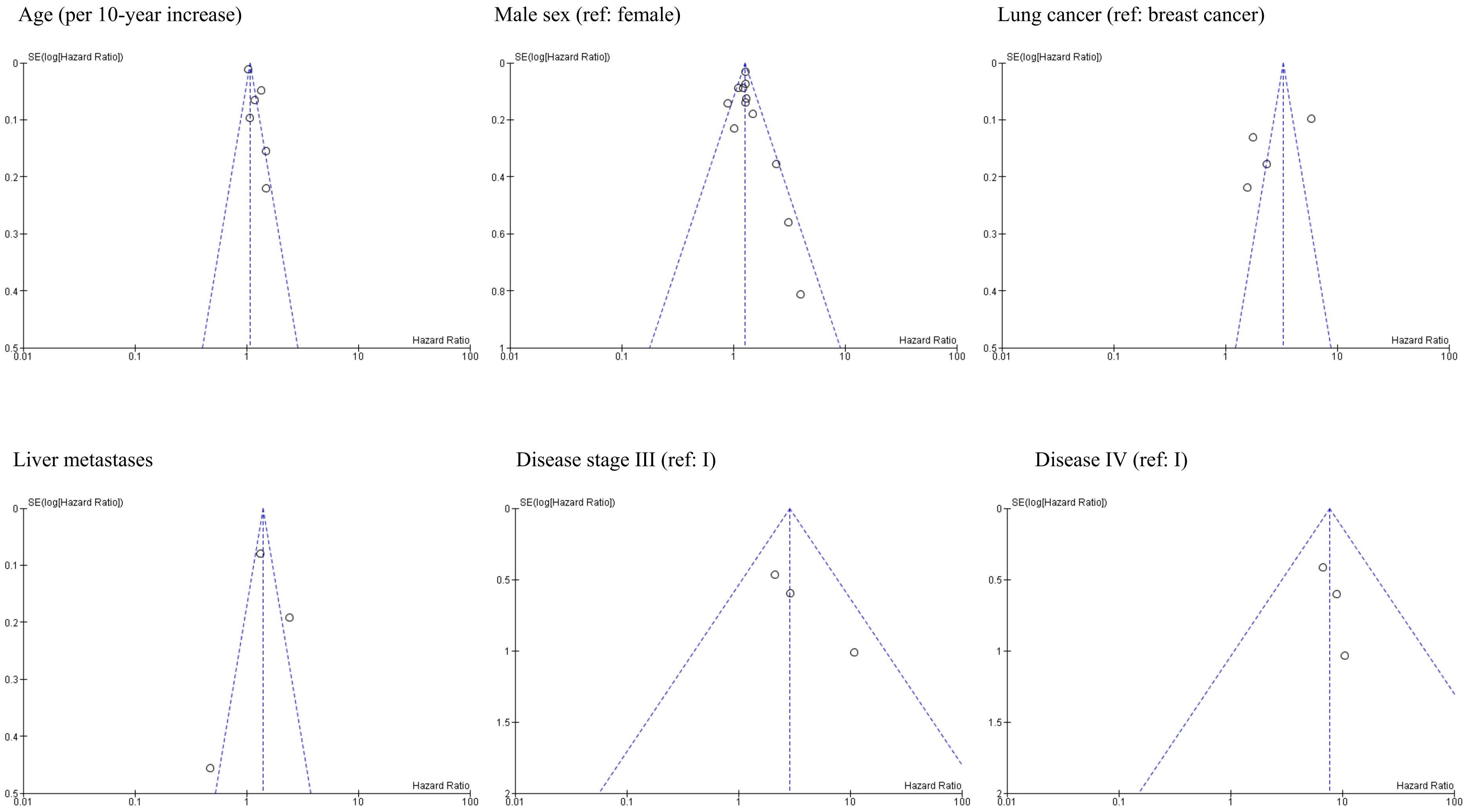

Continuation Figure S2

Karnofsky performance status

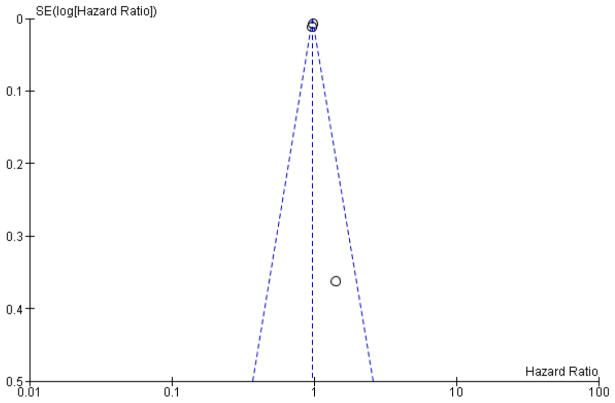

ECOG performance status 1 (ref: 0)

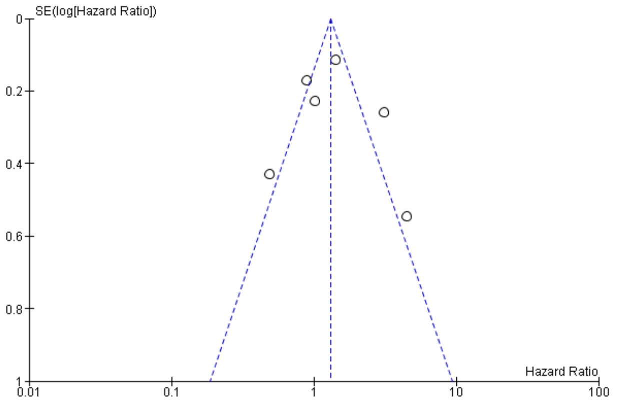

ECOG performance status 1+ (ref: 0–1)

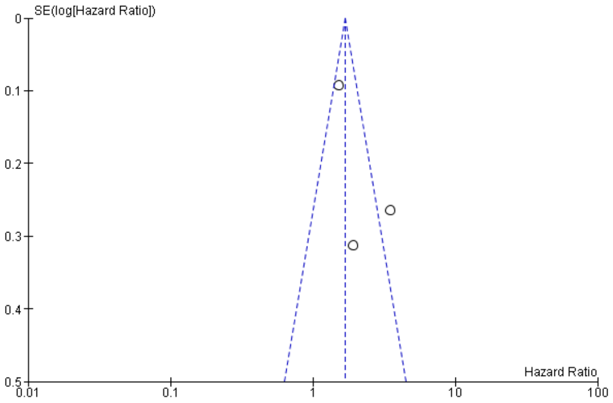

ECOG performance status 1–2 (ref: 0)

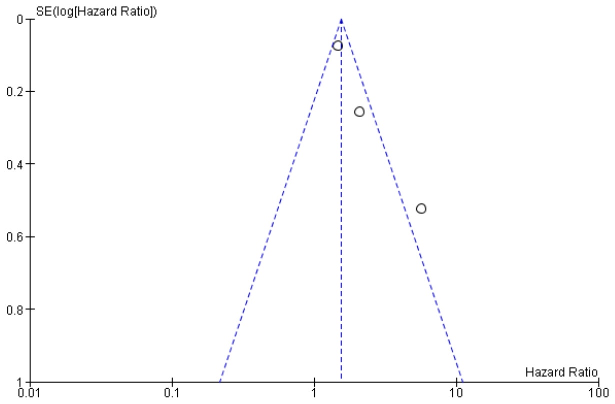

ECOG performance status 2 (ref 0)

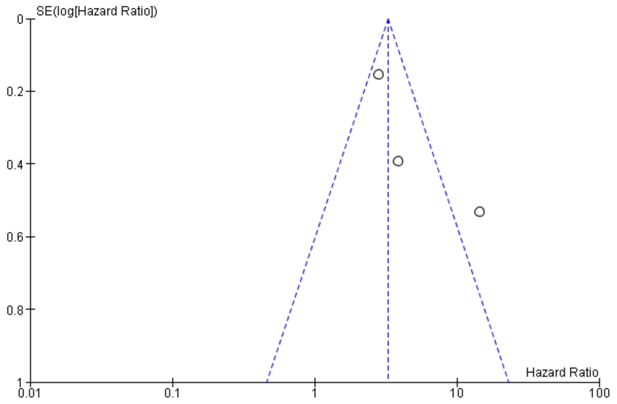

ECOG performance status 2+ (ref: 0)

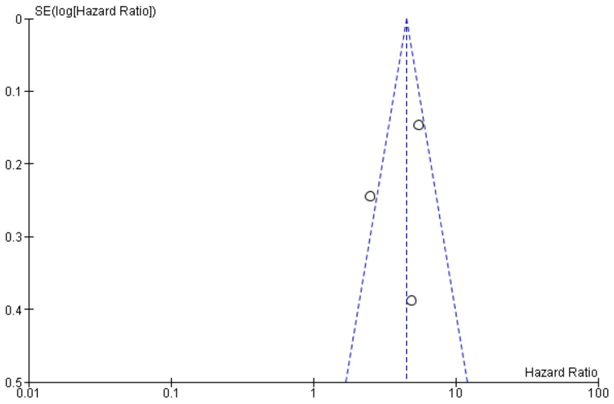

Continuation Figure S2

ECOG performance status 2 (ref: 0–1)

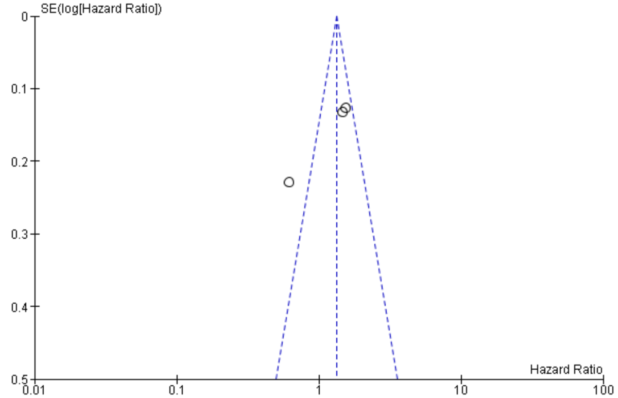

ECOG performance status 3–4 (ref: 0–1)

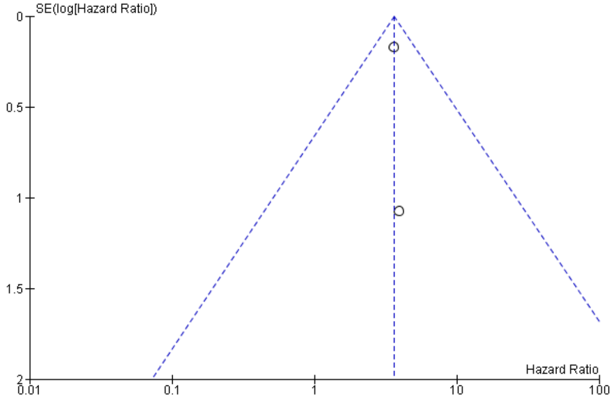

Surprise question ‘no’ (ref: ‘yes’)

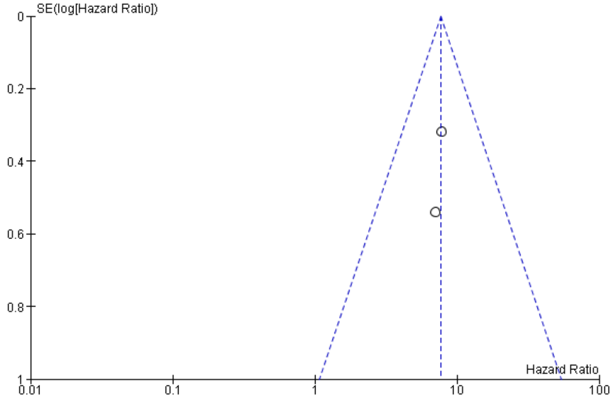

Body mass index (kg/m^2), per unit increase

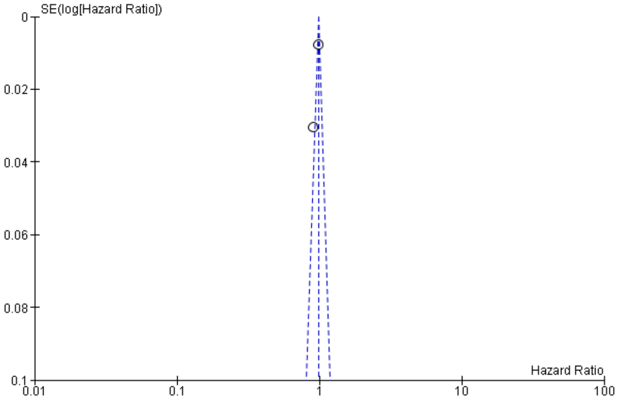

(Sub)cutaneous metastasis

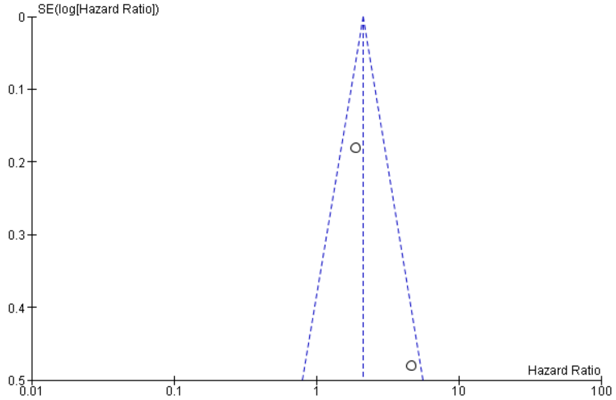

Charlson comorbidity score 1–2 (ref: 0)

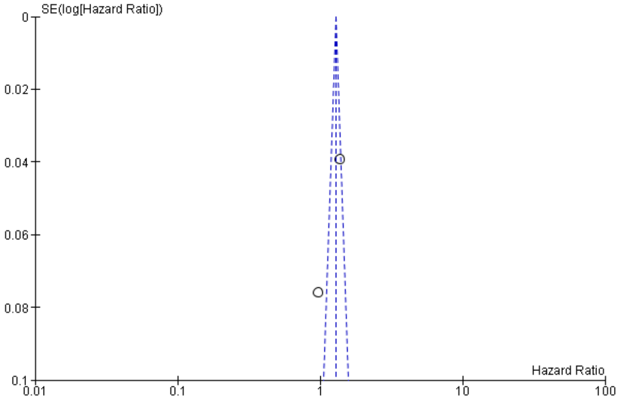

Continuation Figure S2

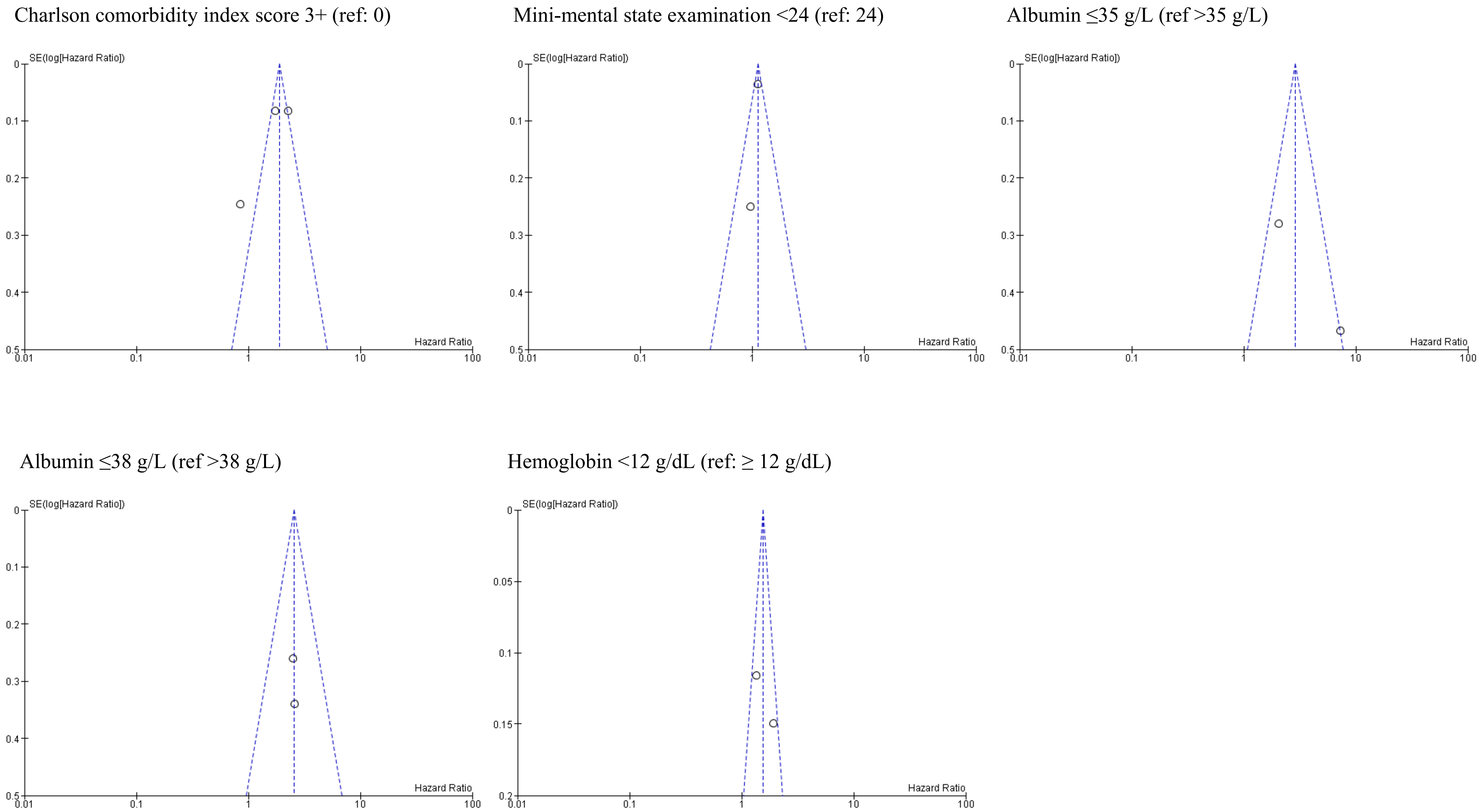

References

1. Filippini G, Falcone C, Boiardi A, Broggi G, Bruzzone MG, Caldiroli D, Farina R, Farinotti M, Fariselli L, Finocchiaro G, et al. Prognostic factors for survival in 676 consecutive patients with newly diagnosed primary glioblastoma. Neuro Oncol. 2008;10(1):79–87.
2. Scott HR, McMillan DC, Forrest LM, Brown DJ, McArdle CS, Milroy R. The systemic inflammatory response, weight loss, performance status and survival in patients with inoperable non-small cell lung cancer. Br J Cancer. 2002;87(3):264–7.
3. Braun DP, Gupta D, Staren ED. Quality of life assessment as a predictor of survival in non-small cell lung cancer. BMC Cancer. 2011;11.
4. Motzer RJ, Bacik J, Schwartz LH, Reuter V, Russo P, Marion S, Mazumdar M. Prognostic factors for survival in previously treated patients with metastatic renal cell carcinoma. J Clin Oncol. 2004;22(3):454–63.
5. Griguolo G, Jacot W, Kantelhardt E, Dieci MV, Bourgier C, Thomssen C, Bailleux C, Miglietta F, Braccini AL, Conte P, et al. External validation of Modified Breast Graded Prognostic Assessment for breast cancer patients with brain metastases: A multicentric European experience. Breast. 2018;37:36–41.
6. Villa S, Weber DC, Moretones C, Manes A, Combescure C, Jove J, Puyalto P, Cuadras P, Bruna J, Verger E, et al. Validation of the new Graded Prognostic Assessment scale for brain metastases: a multicenter prospective study. Radiat oncol. 2011;6:23.
7. Katagiri H, Takahashi M, Wakai K, Sugiura H, Kataoka T, Nakanishi K. Prognostic factors and a scoring system for patients with skeletal metastasis. J Bone Joint Surg Br. 2005;87(5):698–703.
8. Orskov M, Iachina M, Guldberg R, Mogensen O, Mertz Norgard B. Predictors of mortality within 1 year after primary ovarian cancer surgery: a nationwide cohort study. BMJ Open. 2016;6(4):e010123.
9. Suzuki H, Ito M, Takemura K, Nakanishi Y, Kataoka M, Sakamoto K, Tobisu KI, Koga F. Prognostic significance of the controlling nutritional status (CONUT) score in advanced urothelial carcinoma patients. Urol Oncol Semin Orig Invest. 2020;38(3):76.e11–76.e17.
10. Maione P, Perrone F, Gallo C, Manzione L, Piantedosi F, Barbera S, Cigolari S, Rosetti F, Piazza E, Robbiati SF, et al. Pretreatment quality of life and functional status assessment significantly predict survival of elderly patients with advanced non-small-cell lung cancer receiving chemotherapy: a prognostic analysis of the multicenter Italian lung cancer in the elderly study. J Clin Oncol. 2005;23(28):6865–72.
11. Rydzek J, Gąsior ZT, Dąbek J, Wojnar J, Skrzypek M. Assessment of risk factors for mortality in patients with cardiovascular disease and a history of treatment for malignancy. Kardiol Pol. 2015;73(9):730–9.
12. Brunello A, Fontana A, Zafferri V, Panza F, Fiduccia P, Basso U, Copetti M, Lonardi S, Roma A, Falci C, et al. Development of an oncological-multidimensional prognostic index (Onco-MPI) for mortality prediction in older cancer patients. J Cancer Res Clin Oncol. 2016;142(5):1069–77.
13. Vigano A, Bruera E, Jhangri GS, Newman SC, Fields AL, Suarez-Almazor ME. Clinical survival predictors in patients with advanced cancer. Arch Intern Med. 2000;160(6):861–8.
14. Seow H, Barbera L, Dudgeon D, Howell D, Husain A, Atzema C, Sussman J, Liu Y, Earle C, Sutradhar R. The association of the palliative performance scale and hazard of death in an ambulatory cancer population. J Palliat Med. 2013;16(2):156–62.
15. Pointillart V, Vital JM, Salmi R, Diallo A, Quan GM. Survival prognostic factors and clinical outcomes in patients with spinal metastases. J Cancer Res Clin Oncol. 2011;137(5):849–56.
16. Bartels RH, Feuth T, van der Maazen R, Verbeek AL, Kappelle AC, Andre Grotenhuis J, Leer JW. Development of a model with which to predict the life expectancy of patients with spinal epidural metastasis. Cancer. 2007;110(9):2042–9.
17. Cesari M, Cerullo F, Zamboni V, Di Palma R, Scambia G, Balducci L, Antonelli Incalzi R, Vellas B, Gambassi G. Functional status and mortality in older women with gynecological cancer. J Gerontol A Biol Sci Med Sci. 2013;68(9):1129–33.
18. Chow E, Abdolell M, Panzarella T, Harris K, Bezjak A, Warde P, Tannock I. Predictive model for survival in patients with advanced cancer. J Clin Oncol. 2008;26(36):5863–9.

19. Collette L, van Andel G, Bottomley A, Oosterhof GO, Albrecht W, de Reijke TM, Fossa SD. Is baseline quality of life useful for predicting survival with hormone-refractory prostate cancer? A pooled analysis of three studies of the European Organisation for Research and Treatment of Cancer Genitourinary Group. J Clin Oncol. 2004;22(19):3877–85.
20. Deans DAC, Wigmore SJ, de Beaux AC, Paterson-Brown S, Garden OJ, Fearon KCH. Clinical prognostic scoring system to aid decision-making in gastro-oesophageal cancer. Br J Surg. 2007;94(12):1501–8.
21. Gripp S, Moeller S, Bolke E, Schmitt G, Matuschek C, Asgari S, Asgharzadeh F, Roth S, Budach W, Franz M, et al. Survival prediction in terminally ill cancer patients by clinical estimates, laboratory tests, and self-rated anxiety and depression. J Clin Oncol. 2007;25(22):3313–20.
22. Marrero JA, Fontana RJ, Barrat A, Askari F, Conjeevaram HS, Su GL, Lok AS. Prognosis of hepatocellular carcinoma: comparison of 7 staging systems in an American cohort. Hepatology. 2005;41(4):707–16.
23. Ueno H, Okada S, Okusaka T, Ikeda M. Prognostic factors in patients with metastatic pancreatic adenocarcinoma receiving systemic chemotherapy. Oncology. 2000;59(4):296–301.
24. Yamashita T, Siemionow KB, Mroz TE, Podichetty V, Lieberman IH. A prospective analysis of prognostic factors in patients with spinal metastases: use of the revised Tokuhashi score. Spine. 2011;36(11):910–7.
25. Jonna S, Chiang L, Liu J, Carroll MB, Flood K, Wildes TM. Geriatric assessment factors are associated with mortality after hospitalization in older adults with cancer. Supportive Care Cancer. 2016;24(11):4807–13.
26. Hosono N, Ueda T, Tamura D, Aoki Y, Yoshikawa H. Prognostic relevance of clinical symptoms in patients with spinal metastases. Clin Orthop Relat Res. 2005(436):196–201.
27. Liljehult MM, Buus L, Liljehult J, Rasmussen BK. Walking ability in patients with glioblastoma: prognostic value of the Berg Balance Scale and the 10 meter walk test. J Neuro-Oncol. 2017;135(2):335–42.
28. Soubeyran P, Fonck M, Blanc-Bisson C, Blanc JF, Ceccaldi J, Mertens C, Imbert Y, Cany L, Vogt L, Dauba J, et al. Predictors of early death risk in older patients treated with first-line chemotherapy for cancer. J Clin Oncol. 2012;30(15):1829–34.
29. Kilgour RD, Vigano A, Trutschnigg B, Lucar E, Borod M, Morais JA. Handgrip strength predicts survival and is associated with markers of clinical and functional outcomes in advanced cancer patients. Supportive Care Cancer. 2013;21(12):3261–70.
30. Dharma-Wardene M, Au HJ, Hanson J, Dupere D, Hewitt J, Feeny D. Baseline FACT-G score is a predictor of survival for advanced lung cancer. Qual Life Res. 2004;13(7):1209–16.
31. Fielding R, Wong WS. Quality of life as a predictor of cancer survival among Chinese liver and lung cancer patients. Eur J Cancer. 2007;43(11):1723–30.
32. Moss AH, Lunney JR, Culp S, Auber M, Kurian S, Rogers J, Dower J, Abraham J. Prognostic significance of the "surprise" question in cancer patients. J Palliat Med. 2010;13(7):837–40.
33. Hoang T, Xu R, Schiller JH, Bonomi P, Johnson DH. Clinical model to predict survival in chemonaive patients with advanced non-small-cell lung cancer treated with third-generation chemotherapy regimens based on eastern cooperative oncology group data. J Clin Oncol. 2005;23(1):175–83.
34. Penel N, Hollebecque A, Maynou C, Dewaele J, Jasserand M, Beuscart R, Vieillard MH. Development of a score that predicts survival among patients with bone metastasis revealing solid tumor. Support Care Cancer. 2008;16(9):1089–93.
35. Pinato DJ, Karamanakos G, Ishizuka M, Smirne C, Pirisi M, Kubota K, Sharma R. The Kings Score refines prognostic prediction in hepatocellular carcinoma: a novel application. Liver Int. 2015;35(11):2458–65.
36. Wei J, Tang D, Nie Y, Chen J, Peng L. Clinical characteristics and prognosis of nonsurgically treated patients with pneumonic-type adenocarcinoma. Medicine. 2019;98(18).
37. Roychowdhury DF, Hayden A, Liepa AM. Health-related quality-of-life parameters as independent prognostic factors in advanced or metastatic bladder cancer. J Clin Oncol. 2003;21(4):673–8.

38. Hong YF, Chen ZH, Ma XK, Li X, Wu DH, Chen J, Dong M, Wei L, Wang TT, Ruan DY, et al. Comparison of five models for end-stage liver disease in predicting the survival rate of patients with advanced hepatocellular carcinoma. Tumour Biol. 2016;37(4):5265–73.
39. Limquiaco JL, Wong GLH, Wong VWS, Lai PBS, Chan HLY. Evaluation of model for end stage liver disease (MELD)-based systems as prognostic index for hepatocellular carcinoma. J Gastroenterol Hepatol. 2009;24(1):63–9.
40. Chen CK, Feng CL, Yu CJ, Peng CY, Lai HC, Cheng KS, Lin YC, Huang WH, Kao JT. Prognostic factors associated with the survival of patients with gastric adenocarcinoma: A retrospective study. Adv Dig Med. 2019.
41. Giantin V, Valentini E, Iasevoli M, Falci C, Siviero P, De Luca E, Maggi S, Martella B, Orru G, Crepaldi G, et al. Does the Multidimensional Prognostic Index (MPI), based on a Comprehensive Geriatric Assessment (CGA), predict mortality in cancer patients? Results of a prospective observational trial. J Geriatr Oncol. 2013;4(3):208–17.
42. Hui D, Bansal S, Morgado M, Dev R, Chisholm G, Bruera E. Phase angle for prognostication of survival in patients with advanced cancer: preliminary findings. Cancer. 2014;120(14):2207–14.
43. Martin L, Watanabe S, Fainsinger R, Lau F, Ghosh S, Quan H, Atkins M, Fassbender K, Downing GM, Baracos V. Prognostic factors in patients with advanced cancer: Use of the patient-generated subjective global assessment in survival prediction. J Clin Oncol. 2010;28(28):4376–83.
44. Tripodoro VA, Llanos V, De Lellis S, Salazar Güemes C, De Simone GG, Gómez-Batiste X. Prognostic factors in cancer patients with palliative needs identified by the necpal CCOMS-ICO© tool. Medicina. 2019;79(2):95–103.
45. Sutradhar R, Atzema C, Seow H, Earle C, Porter J, Barbera L. Repeated assessments of symptom severity improve predictions for risk of death among patients with cancer. J Pain Symptom Manage. 2014;48(6):1041–9.
46. Efficace F, Bottomley A, Smit EF, Lianes P, Legrand C, Debruyne C, Schramel F, Smit HJ, Gaafar R, Biesma B, et al. Is a patient's self-reported health-related quality of life a prognostic factor for survival in non-small-cell lung cancer patients? A multivariate analysis of prognostic factors of EORTC study 08975. Ann Oncol. 2006;17(11):1698–704.
47. Kinoshita A, Onoda H, Takano K, Imai N, Saeki C, Fushiya N, Miyakawa Y, Nishino H, Tajiri H. Pretreatment serum C-reactive protein level predicts poor prognosis in patients with hepatocellular carcinoma. Med Oncol. 2012;29(4):2800–8.
48. Penel N, Vanseymortier M, Bonneterre ME, Clisant S, Dansin E, Vendel Y, Beuscart R, Bonneterre J. Prognostic factors among cancer patients with good performance status screened for phase I trials. Invest New Drugs. 2008;26(1):53–8.
49. Park HS, Lee HS, Park JS, Park JS, Lee DK, Lee SJ, Yoon DS, Lee MG, Jeung HC. Prognostic Scoring Index for Patients with Metastatic Pancreatic Adenocarcinoma. Cancer Res Treat. 2016.
